# Selenium-Related Transcriptional Regulation of Gene Expression

**DOI:** 10.3390/ijms19092665

**Published:** 2018-09-08

**Authors:** Mikko J. Lammi, Chengjuan Qu

**Affiliations:** 1 Key Laboratory of Trace Elements and Endemic Diseases, National Health and Family Planning, Institute of Endemic Diseases, School of Public Health of Health Science Center, Xi’an Jiaotong University, Xi’an 710061, China; 2 Department of Integrative Medical Biology, University of Umeå, 901 87 Umeå, Sweden; chengjuan.qu@umu.se

**Keywords:** selenium, selenocysteine, selenoproteins, selenocysteine insertion sequence, nonsense-mediated decay

## Abstract

The selenium content of the body is known to control the expression levels of numerous genes, both so-called selenoproteins and non-selenoproteins. Selenium is a trace element essential to human health, and its deficiency is related to, for instance, cardiovascular and myodegenerative diseases, infertility and osteochondropathy called Kashin–Beck disease. It is incorporated as selenocysteine to the selenoproteins, which protect against reactive oxygen and nitrogen species. They also participate in the activation of the thyroid hormone, and play a role in immune system functioning. The synthesis and incorporation of selenocysteine occurs via a special mechanism, which differs from the one used for standard amino acids. The codon for selenocysteine is a regular in-frame stop codon, which can be passed by a specific complex machinery participating in translation elongation and termination. This includes a presence of selenocysteine insertion sequence (SECIS) in the 3′-untranslated part of the selenoprotein mRNAs. Nonsense-mediated decay is involved in the regulation of the selenoprotein mRNA levels, but other mechanisms are also possible. Recent transcriptional analyses of messenger RNAs, microRNAs and long non-coding RNAs combined with proteomic data of samples from Keshan and Kashin–Beck disease patients have identified new possible cellular pathways related to transcriptional regulation by selenium.

## 1. Introduction

Selenium is a trace element and a vital nutrient component. It is present in various forms, such as inorganic sodium selenate and sodium selenite and, for instance, also as selenomethionine, selenocysteine, y-glutamyl-selenium-methylselenocysteine, selenium-methyl selenocysteine and methylselenol [1,2]. These various forms have different oxidation states: +6 in selenates, +4 in selenites, 0 in elemental selenium, and −2 in inorganic and organic selenides [3], which affect their bioavailability and properties. In food, selenium is mainly associated with protein in animal tissue, particularly in meat and seafood, but also in bread and cereals [1,2]. In plants, selenium is converted to methylated selenium components, selenomethione and selenocysteine (Sec) [4,5]. There are also many enteral formulas supplemented with selenium, too [2].

The major bioavailable forms of selenium are organic forms, such as Sec and selenomethionine, and inorganic selenate and selenite. A recommended average daily intake for individuals over 14 years of age is 55 µg according to the Office of Dietary Supplements of the National Institutes of Health (Bethesda, MD, USA), while the need in younger children ranges between 15–40 µg/day. Globally, the dietary intake can often be below that recommendation [6], and there may be even one billion people affected by selenium deficiency, mainly due to a low selenium content in the soil. Geographical variation in the soil selenium contents occurs globally, and especially in China the soil shows highly variable contents [7].

The first findings related to selenium were those noted on its toxicity already in 19th century. The symptoms of selenium toxicity include fatigue, and disturbances in connective tissue, cardiovascular, nervous, gastrointestinal and respiratory systems [8]. Selenosis due to unusually high concentrations of dietary selenium leads also to poor dental health, brittle hair and nails, nausea, vomiting and pulmonary oedema, the symptom severity depending on the level of poisoning [9]. In addition, selenium can even interact with arsenic, increasing the toxicity [10].

Many chronic diseases are also related to decreased selenium status. Increased incidences of myocardial infarction and death have been noticed to be associated with selenium deficiency [11]. Low selenium has also been related to cancer, renal diseases and viral infection [4]. Chinese endemic diseases, such as the Keshan and Kashin–Beck diseases are mainly observed in a geographic belt located from northeast to southwest in China with a very low content of water-soluble selenium [12]. Selenium deficiency is also associated with fibrosis of various organs, such as heart, liver, kidney, thyroid and pancreas, and both to fibrotic cystic and oral submucosa [13].

The supplementation of selenium in foodstuff or fertilizers to achieve adequate supply has been practiced in variable ways. In the 1970s, Finland was among those countries that had the lowest selenium levels in the population. Since Finland also had a high incidence of cardiovascular diseases, an association of these factors was hypothesized. A large-scale fortification of fertilizers supplemented with selenium increased the selenium contents of bread and milk in Finland so that the selenium levels in the serum almost doubled in the population [3]. Patients with viral or bacterial infections may benefit from dietary selenium supplementation [14].

The preventive and therapeutic efficiency of selenium in cancer depends on the form of selenium [15]. It has been noticed that four-valent sodium selenite can specifically oxidize the vicinal sulfhydryl groups [16,17], in contrast to six-valent selenate [16]. It has been found that hydrophobic polymers coating the plasma membrane can make the cells resistant to the recognition and destruction by the innate immune system, while redox-active sodium selenite can inhibit this process by unmasking specific tumor antigens, which upon immune recognition allow natural killer cells to eliminate the tumor cells [18]. Importantly, sodium selenite can also directly activate the natural killer cells [19]. Besides solid tumors [20], selenite appears to have an anti-leukemic effect [21], and to be potentially useful for the treatment of multidrug-resistant acute myeloid leukemia [22].

In this review, we briefly discuss the functions of the selenoproteins, the events related to selenoprotein biosynthesis, and how selenium deficiency affects the gene regulation of selenoproteins, but also other genes. The role of long non-coding RNAs (lncRNAs) is also handled, while the focus of this review is on human and other mammalian observations.

## 2. Selenoprotein Functions and Selenoprotein-Related Disorders

Selenium is incorporated into proteins mainly as Sec, the 21st amino acid [23]. There are 24 selenoproteins identified in rodents and 25 in humans [24]. These include five glutathione peroxidases, three iodothyronine deiodinases, two thioredoxin reductases, thioredoxin-glutathione reductase 3, selenophosphate synthetase 2, methionine sulfoxide reductase B1, and selenoproteins F, H, I, K, M, N, O, P, S, T, V and W [25]. The best-known selenoproteins are glutathione peroxidases, thioredoxin reductases, iodothyronine deiodinases and selenoprotein P.

Selenoprotein biosynthesis is essential for life, since deletion of the murine gene encoding selenocysteine tRNA, which inserts selenocysteine to a growing polypeptide during translation, led to an early embryonic lethality [26]. The functions of many of the selenoproteins have been identified so far, although there are still those with unknown functions [27]. Almost all of them are oxidoreductases, and they have been localized to plasma membrane, endoplasmic reticulum, cytosol, mitochondria, and nucleus, and even two secreted extracellular selenoproteins are present [27]. They also have diverse patterns of tissue distribution, which can vary from ubiquitous to very tissue-specific locations [28]. The selenoproteins involve antioxidant and redox reactions by the detoxification of peroxides, a regeneration of reduced thioredoxin and a reduction of oxidized methionine residues, and iodothyronine deiodinases regulate the activity of thyroid hormones [28]. Some selenoproteins are also involved in calcium mobilization, selenium transport and endoplasmic reticulum stress [28].

Universal genetic code has three codons reserved for translation termination. Of those, the UGA codon also signals for the incorporation of Sec into the selenoproteins [29]. A Sec insertion sequence (SECIS), which is a specific stem–loop structure in the 3′-untranslated regions (UTRs) of the messenger RNAs (mRNAs) in eukaryotes, makes the cotranslational incorporation of Sec into nascent polypeptides possible [30]. The mammalian selenoproteins usually contain a single Sec residue located in the enzymes’ active site, with the exception of the selenoprotein P, which has multiple residues [31]. The presence of Sec in the active site of the selenoproteins is important for its activity, and a misinterpretation of the UGA codon can lead to a significant loss of function of the selenoprotein. Still, many selenoproteins have functional orthologs, which have cysteine occupying the position of Sec [32], although they usually have poor activity in comparison to the Sec-containing selenoprotein.

Mutations and inborn errors in the genes involved in selenoprotein biosynthesis have been identified [33]. The first selenoprotein-related mutations were observed in the selenoprotein N [34], and it has been noticed that its mutations led to a spectrum of myopathies. Other disorders include neurologic phenotypes due to, for instance, the selenium deficiency in the brain caused by impaired selenium transport in the selenoprotein P-deficient mice [35,36]. The mutations in the glutathione peroxidase 4 cause Sedaghatian-type spondylometaphyseal chondrodysplasia [37], and those in thioredoxin system may affect the heart and the adrenals [38,39]. There are no known mutations of iodothyronine deiodinases in humans, but in mice the deletion of iodothyronine deiodinase 2 impaired bone stability [40]. The mutations in SECIS-binding protein 2 gene affect thyroid hormone regulation [41], but can have variable other features as well. The first identified human mutation in selenocysteine transfer RNA (tRNA^[Ser] Sec^) manifested a similar phenotype as mutations in SECIS-binding protein 2 gene [42]. In mice, deletion of the gene encoding the tRNA^[Ser] Sec^ in osteochondroprogenitor yielded a phenotype similar to Kashin–Beck disease [43], although the phenotype is clearly stronger than the one observed in human Kashin–Beck disease [44].

## 3. Biosynthesis of Selenocysteine and Selenoproteins

There are two principal elements in the biosynthesis of selenoproteins and recoding of the UGA stop codon [45]. These are the involvement of (1) tRNA^[Ser] Sec^, which has an anticodon to the UGA codon, and (2) SECIS in the 3′-UTR. tRNA^[Ser] Sec^, first described in 1970 [46,47], is a unique tRNA in being able to regulate a whole class of proteins, namely the selenoproteins.

The aminoacylation of tRNA^[Ser] Sec^ differs remarkably from the other tRNAs, since it requires the action of four different enzymes instead of only the one needed for the others [45]. In eukaryotes, the synthesis of Sec is started so that serine aminoacyl tRNA synthetase charges tRNA^[Ser] Sec^ with serine. In the next step, tRNA^[Ser] Sec^ serine is phosphorylated by a specific phosphoseryl tRNA kinase and, finally, the phosphate of *O*-phosphoserine of the tRNA^[Ser] Sec^ is substituted by selenium atom donated by selenophosphate in a reaction catalyzed by selenocysteine synthase [45]. Selenophosphate synthetase 2 is essential in mammals to generate selenophosphate, while a highly homologous selenophosphate synthetase 1 has only little or no effect on selenoprotein synthesis [48,49].

Every selenoprotein mRNA contains a stem–loop–stem–loop structure in the 3′-end of their mRNA, which is an approximately 200 nucleotides long sequence. They are essential for the recoding of the UGA codons during selenoprotein translation. It has been noticed that the SECIS motif is in fact important for the modulation of the efficiency of Sec insertion, so that several thousand-fold differences can be observed between the strongest and weakest SECIS elements [50]. The minimum distance of the SECIS motif from the UGA codon is evaluated to be 51–111 nucleotides [51].

There are many proteins involved in the recoding of Sec. The best characterized of those is SECIS-binding protein 2 (SBP2) [52], which is found in all eukaryotes expressing selenoproteins. It is one of the limiting factors for Sec insertion [53], and its silencing caused a selective down-regulation of the selenoprotein expression in the mammalian cells [54]. It has been shown that SBP2 binding affinity also has a major importance for differential selenoprotein mRNA translation and sensitivity to so-called nonsense-mediated decay (NMD) [55]. The cells use a complex NMD pathway to destroy mRNAs with the premature stop codon to prevent their translation, which would lead to a synthesis of incorrect proteins [56]. Translation initiation factor 4A3 (eIF4A3) is a SECIS-binding protein, which takes part in a selective translational control of a subset of the selenoproteins [57]. Its binding to a subset of SECIS elements physically limits the binding of SBP2 there, thus preventing Sec insertion [57], and leads to the premature stop of translation. It has been shown that approximately half of the selenoprotein transcriptome has a SECIS element susceptible to NMD, which leads to remarkable changes of the transcript levels under selenium deficiency [58].

Ribosomal protein L30 is a SECIS-binding protein as a component of the large unit of eukaryotic ribosome, and can apparently recruit SBP2 to the Sec recoding machinery [59], but its exact function in Sec insertion needs further investigation. A multifunctional protein, nucleolin, is another SECIS-binding protein, which is likely to functions in Sec translation complex as a link to the ribosome [60].

## 4. Regulation of Selenoprotein Transcriptome by Selenium

Selenium intake has been observed to significantly affect the selenoproteome. During selenium deficiency, the gene expression and production of certain obviously essential selenoproteins is prioritized over others [61,62]. In a one-day-old layer of chicken liver, a prolonged deficiency of selenium in the diet continued for up to 65 days decreased 19 selenoprotein mRNA expressions of 21 investigated ones [63]. However, the sensitivities of various selenoprotein gene expressions to decrease was differential [63]. In general, interpretations of the results accumulated from the published studies are somewhat complicated, since the tissue distribution of the specific selenoproteins and their various intra- and extracellular locations is variable [27,64].

A considerable number of the selenoproteins participate in glutathione and thioperoxin systems, which play important roles in maintaining cellular redox balance [65]. Glutathione peroxidases are catalysts for the reduction of H_2_O_2_ or organic hydroxyperoxides to water or corresponding alcohols, most often by using glutathione as a reductant. Their dysregulation has been associated with diabetes, cancer, inflammation, and obesity [66]. Glutathione is one of the most important intracellular antioxidant systems, although it is also present in extracellular environments [67]. Thus, it acts as the major redox buffer in the majority of the cells. Glutathione in its reduced form can scavenge reactive oxygen and nitrogen species, a process which yields oxidized glutathione disulfide [65]. An enzyme glutathione reductase recycles oxidized glutathione back to its reduced form using nicotinamide adenine dinucleotide phosphate as a universal reducing agent [68]. Besides this system, glutathione metabolism involves numerous other proteins and electrophiles [69].

During aging, the redox balance of our body is jeopardized by oxidative stress, which is indicated by the oxidation of, for instance, glutathione, which is also related to our health and disease [70]. Selenoglutathione is a selenium analogue of glutathione, and a biologically potent redox substrate. Its oxidized form was recently shown to have the potential to repair misfolded disulfide-bond-containing proteins [71]. Since diselenide bonds can be expected to be more stable than disulfide ones, selenoglutathione analogues have also raised medical interest as possibly effective antioxidative agents [72].

Thioredoxin is a highly conserved antioxidant protein, which also participates in the maintenance of the redox balance by thiol-disulfide exchange reactions [73]. The thioredoxin system acts together with glutathione, and they can provide the electrons that cross-control the cellular redox environment [74].

### 4.1. Glutathione Peroxidases

Five of the glutathione peroxidases in humans are selenoproteins. Ubiquitously present glutathione peroxidase 1 was the first identified selenoprotein, which was then found to protect hemoglobin from oxidative breakdown [75]. Its gene expression and enzyme activity was almost totally lost in the liver and the heart under the depletion of selenium in the diet of rats, while in glutathione peroxidase 4, also known as phospholipid hydroperoxide glutathione reductase, gene expression was practically unchanged despite the 75% and 60% decrease of enzymatic activity, respectively [64].

In rats, glutathione peroxidase 2 was observed to be expressed only in the epithelium of the gastrointestinal tract, therefore, it is also called gastrointestinal glutathione peroxidase [76]. Its expression has also been reported for the human liver [77]. The selenium deficiency even appeared to increase the gene expression of glutathione peroxidase 2, while glutathione peroxidase 1 levels dropped and glutathione peroxidase 4 levels remained unchanged [78].

Glutathione peroxidase 3 is an abundant plasma protein, which is mostly secreted by kidney proximal tubules [79]. Studies with double knock-out mice for the glutathione peroxidase 3 and the selenoprotein P showed that together these two proteins account for the majority (>97%) of all plasma selenium in mice [80]. 

Besides H_2_O_2_, glutathione peroxidase 4 can also reduce the hydroperoxides present in phospholipids and cholesterol [66]. It is present in three different isoforms, namely the cytosolic, mitochondrial and sperm nuclear forms [81]. Their expression levels have been shown to be relatively resistant to the selenium deficiency [54,64], and therefore they are considered to be among the essential selenoproteins [82].

### 4.2. Thioredoxin Reductases

Humans and higher eukaryotes have three thioredoxin reductases, namely cytosolic, mitochondrial and testis-specific [83], which all contain the in-frame UGA codon to encode Sec residue. Although the mutation of Sec to Cys leads to a major decrease in their catalytic activity, Sec is not catalytically essential to reduce thioredoxin due to orthologs, which can perform identical reactions with approximately the same efficiencies [84]. Thioredoxin reductase 1 has been shown to be different from thioredoxin reductase 3 by containing a C-terminal structure, which restricts the motion of the C-terminal tail containing Gly-Cys-Sec-Gly tetrapeptide so that only that C-terminal redox center can participate with Se-dependent reduction by the N-terminal redox center, while thioredoxin reductase 3 does not have that same structure [85]. Therefore, thioredoxin reductase 3 has a greater access to the substrate and an alternative mechanistic pathway [85].

The selenium deficiency was shown to decrease the expression levels of both thioredoxin reductases 1 and 2 in rat heart, as well as glutathione reductases 1 and 4, which is known to impair the recovery from ischemia-reperfusion, while selenium supplementation significantly increased their expression levels [86]. Although glutathione peroxidases and thioredoxin reductases have been shown to be similarly modulated by the selenium availability in cardiac tissue [86], and to cooperate in the protection against free-radical-mediated cell death [87], they were oppositely regulated by selenium in response to replicative senescence in human embryonic fibroblasts [88].

Thioredoxin reductase 1 is overexpressed in many malignant cells and thus it has been considered as a target for anticancer approaches [89]. The transcriptional regulation of human thioredoxin reductase is very complex [90]. It has at least 21 different transcripts, which encode five isoforms differing in the alternative N-terminal domains [91]. It has been observed that selenium overdose can have selective cytotoxic effects on tumor cells [92].

### 4.3. Iodothyronine Deiodinases

Type 1 iodothyronine deiodinase produces biologically active 3,5,3′-triiodothyronine (T3) from 3,5,3′,5′-tetraiodothyronine (T4, or thyroxin) for the plasma, while type II enzyme provides a local conversion. Type 3 enzyme finally converts T3 to inactive T2. All three deiodinases are expressed in a number of fetal and adult tissues [93]. The finding that selenium deficiency inhibits the activity of type I and II iodothyronine deiodinases led to the proposal that they are seleno-enzymes or selenium-containing cofactors [94], and type I iodothyronine deiodinase was soon identified as a selenoenzyme [95]. However, the gene expressions of type I iodothyronine deiodinase and selenoprotein P were more resistant to selenium deficiency-related decline than glutathione reductase [96]. In chicken, type I deiodinase expression was the most sensitive to prolonged selenium deficiency, while the type III enzyme did not change significantly even after 65 days deprivation [63]. Thyroid is much more resistant to dietary deficiency than, for instance, the liver, indicating the dependency on the organ in terms of how selenium deprivation affects the gene expression of the selenoproteins [97]. In general, the thyroideal conversion of T4 to T3 by iodothyronine deiodinases can be considered to be their major function. However, they have importance in other tissues as well, since type I iodothyronine deiodinase transcripts could be found at even higher levels than the thyroid in the ovine skeletal muscle, kidney and heart [98].

### 4.4. Other Selenoproteins

The selenium availability changes the gene expressions of a number of other selenoproteins, too. In chicken, selenoprotein I was the only one, which did not show a significant decrease at mRNA levels even after 65 days of selenium deficiency, while all the other investigated ones had decreased levels already after 15 days of treatment [63].

Selenoprotein P is unique among the selenoproteins, since it incorporates several Sec residues instead of the normal content of one Sec per one protein molecule. The human selenoprotein contains 10 Sec residues [99]. The liver takes up selenium and incorporates it into selenoprotein P, which is then secreted into the plasma for transport to other tissues [35]. In selenoprotein P knockout mice, a low selenium diet decreased the selenium content most severely in the brain and the testis, while the heart was not affected [36]. Due to its selenium transport characteristics, selenoprotein P also has a special role in the regulation of other selenoproteins.

Selenophosphate-synthetase 2 is an enzyme that participates the biosynthesis of selenoproteins by providing the selenophosphate needed in the biosynthesis of Sec. Thus, it can regulate its own production besides the production of other selenoproteins [100]. In selenoprotein P-depleted mice, all the other selenoproteins were down-regulated in the brain and the testis, in contrast to elevated levels of selenophosphate-synthetase 2 [101], which can obviously provide a compensatory mechanism during low selenium conditions.

A recent selenoproteome transcriptome study showed that the selenium deficiency in an ATDC chondrocyte cell line most dramatically dropped the expression levels of glutathione peroxidase 1 and selenoproteins H, I and W, while selenophosphate synthetase 2 and selenoproteins O, R and S were in fact upregulated [102]. The gene expression levels of glutathione peroxidase 1, thioredoxin reductase 1 and selenoproteins H, P, R and W were the most efficiently increased after the repletion of selenium to selenium-deficient medium [103]. Similar results were obtained for another chondrocyte cell line C28/I2 [103]. A microarray analysis showed that nine selenoproteins were significantly down-regulated in the selenium-deficient liver, while in mice with marginal selenium status none of these transcripts remained decreased relative to the levels in selenium-adequate mice [104]. This was concluded to mean that the selenium-related regulation of the selenoprotein gene expression is mediated by one underlying mechanism [62].

Microarray data of more than 30,000 transcripts from the livers of rats fed with a 0–5 μg selenium/g diet revealed that selenium deficiency down-regulated four selenoprotein genes (glutathione peroxidase 1, thioredoxin reductase 3, and selenoproteins H and T). In mouse, thioredoxin reductase 2 and selenoproteins K and W, muscle 1 were also down-regulated in the liver, and selenoprotein W and muscle 1 in the kidney [102].

## 5. Regulation of Non-Selenoprotein Transcriptome by Selenium

Selenium deficiency also regulates the expression of genes other than those encoding for the selenoproteins. Although limited in number, the recent large-scale transcriptome and proteome analyses have given new insights into the general level effects of selenium deprivation.

A study investigating the effects of various levels of selenium in the diet was continued for 35 days in mice and 28 days in rats [102]. The microarray analysis of the livers of animals fed with 0–5 µg selenium/g diet revealed only two transcripts (uridine diphosphate-glucuronosyltransferase 2 family, polypeptide B7 and glutathione S-transferase Yc2 subunit), which were up-regulated in the rat liver [102], whereas carbonyl reductase 3 and heat shock protein 1 in the mouse liver and glutathione 5-transferase, alpha 3 in the mouse kidney were also induced [102]. Real-time polymerase chain reaction analyses of the rat liver also indicated that adenosine triphosphate-binding cassette, subfamily C (cystic fibrosis transmembrane conductance regulator/multidrug-resistance-related protein), member 3 and nicotinamide dinucleotide phosphate dehydrogenase, quinone 1 were upregulated genes [102], which are the targets of the nuclear factor erythroid-2 like factor 2 (Nrf2) [105]. Since Nrf2 has a major role in the regulation of the cellular antioxidant response [106], and plays a critical role in cancer prevention, this pathway may be associated with the anti-cancer properties of selenium.

In the range of 0.08 to 0.8 μg selenium/g diet there were no remarkable differences in the gene expressions, while 2 μg selenium/g diet upregulated the following transcripts: regulator of G-protein signaling 4, coiled-coil domain containing 80, RGD1560666, 3-oxoacid CoA transferase 1, and expressed sequence tag UI-R-BS1-ayq-f-06-0-UI.s1. The only down-regulated one was cold-inducible RNA binding protein in comparison to a selenium adequate diet [102].

The biggest number of changes in gene expression became evident at selenium levels considered already toxic. There were 1193 transcripts, which had the significantly changed expressions [102]. The rats fed with a 5 μg selenium/g diet had retarded growth and signs of liver damage markers. Therefore, in order to more reliably reveal the selenium-related changes, the transcripts were filtered against the changed transcripts in calorie-restricted rats [105] and those overlapping with Affymetrix’s Rat ToxFX 1.o array to remove the transcripts probably related to growth and toxicity. Duplicate transcripts were also removed. This reduced the number of the transcripts regarded to be most likely selenium-specific to 667 unique transcripts [102]. Gene ontology analyses revealed a number of biological processes related to cellular movement and morphogenesis, extracellular matrix and cytoskeleton organization, and development and angiogenesis affected by the toxic level of selenium [102].

## 6. Selenium Regulation of MicroRNAs and Long Non-Coding RNAs

Besides mRNAs, the cells express groups of non-protein-coding transcripts, which can regulate a wide array of protein coding genes. MicroRNAs (miRNAs) and lncRNAs are two major families of these. In an intestinal cell line, selenium deficiency changed the expression of 50 genes and twelve miRNAs. Pathways related to, for instance, arachidonic acid metabolism, glutathione metabolism, oxidative stress, and mitochondrial respiration were found to be selenium-sensitive. Besides, thirteen transcripts were predicted to be targets for selenium-sensitive miRNAs, three of which were recognized by miR-185. More importantly, the silencing of miR-185 increased glutathione peroxidase 2 and selenophosphate synthetase 2 [107]. In a recent study, several miRNAs were predicted as putative regulators of various glutathione peroxidases [108].

In hepatocarcinoma cell line, it was noticed that miRNA-544a interacted with selenoprotein K, suppressing its expression, and the selenium treatment was able to modulate miR-544a expression [109]. On the other hand, selenium increased the expression level of miR-125a, and the overexpression of miR-125a could inhibit cadmium-induced apoptosis in LLC-PK1 cells [110]. In selenium-deficient rats, cardiac dysfunction was mainly associated with five up-regulated miRNAs (miR-374, miR-16, miR-199a-5p, miR-195 and miR-30e) and three down-regulated ones (miR-3571, miR-675 and miR-450a) [111]. In Caco-2 human adenocarcinoma cells, ten selenium-sensitive miRNAs were identified, which are involved in the regulation of 3588 mRNAs. Pathway analysis indicated that they participate in pathways such as the cell cycle, the cellular response to stress, the canonical Wnt/β-catenin, p53 and mitogen-activated kinase signaling pathways [112].

Only two publications that investigated the regulation of lncRNAs by selenium could be found. The first one investigated lncRNAs in the vascular exudative diathesis of chicken, induced by selenium deficiency. A total of 635 differentially-expressed lncRNAs of 15412 detected ones were identified, and gene ontology analysis suggested an importance of redox in particular. The study could verify that 19 target mRNAs of 23 lncRNAs were related to the redox process [113].

The other study investigated the role of lncRNAs in the selenium deficiency-induced muscle injury in chicken. The study identified 38 lncRNAs and 687 mRNAs that were affected by selenium deficiency. Pathway analyses revealed dysregulated pathways associated with phagosomes, cardiac muscle contraction, and peroxisome proliferator-activated receptors (PPAR) in the selenium-deficient group. In particular the study showed a relationship between lncRNA ALDBGAL0000005049 and stearoyl-CoA desaturase in the PPAR pathway. The down-regulation of that lncRNA led to inflammation by regulating stearoyl-CoA desaturase gene expression [114].

There is still a limited number of studies on non-protein-coding RNAs and selenium, and future investigations will certainly shed new light on how they are involved in selenium-related gene expression. The present studies already show that they are potential regulators of cellular responses and gene expressions.

## 7. Selenium-Related Gene Expressions in Some Pathological Conditions

Selenium has been associated with a number of pathological conditions. Although the mechanism for how selenium is related to pathogenesis is not always very precisely known, new research results may provide new ideas to understand selenium-related diseases.

Keshan disease is an endemic cardiomyopathy related to the selenium deficiency leading to a high mortality rate. To understand better the molecular mechanisms of Keshan disease pathogenesis, a proteomic screening of peripheral blood sera was performed with mass spectrometry to identify differentially-expressed selenium- and zinc-related proteins and their pathway and networks associated with Keshan disease [115]. In total, nineteen selenium- and three zinc-associated proteins were identified among 105 differentially-expressed proteins [115]. Pathway analysis revealed 52 pathways, of which hypoxia-inducible factor-1α and apoptosis pathways were likely to play a role in the selenium-associated functions [115]. A study combining a custom-made microarray, containing 78 probes previously differentially expressed genes in Keshan disease, analyses of peripheral blood mononuclear cell RNA samples obtained from 100 Keshan disease patients and 100 normal controls, and mass spectrometric proteomic analyses of peripheral blood sera of Keshan patients and controls indicated that there are numerous functional pathways and cellular systems associated with the differentially expressed genes and proteins [116]. The limitations of these studies are the fact that the selenium status of the individuals was not known. 

Selenium deficiency has also been associated to Kashin–Beck disease, an endemic osteochondropathy. The differentially expressed genes in the transcriptome analyses comparing cartilage samples from Kashin–Beck disease versus normal control [117] or versus osteoarthritis [118] were other than the selenoproteins. Pathways related to reactive oxygen species and vascular endothelial growth factor were significantly elevated in osteoarthritis compared with Kashin–Beck disease, while the expression of genes of collagen- and nitric oxide-related pathways were elevated in Kashin–Beck disease [119]. Analysis of mitochondria-related genes in Kashin–Beck disease chondrocytes versus normal control identified nine up-regulated genes, which involved three canonical pathways: (1) oxidative phosphorylation; (2) apoptosis signaling; and (3) pyruvate metabolism [120]. In addition, microarray expression profiles of the cultured control and the Kashin–Beck disease chondrocytes revealed 232 up- and 427 down-regulated mRNAs and 316 up- and 631 down-regulated lncRNAs in Kashin–Beck disease patients’ chondrocytes [121]. A lncRNA-mRNA correlation analysis yielded 509 coding–noncoding gene co-expression networks, and eleven lncRNAs were predicted to have *cis*-regulated target genes, and co-expressed mRNAs and lncRNAs formed a large network associated with biological events of the extracellular matrix [121].

The problem with investigations of Kashin–Beck disease is that the disease often develops already in early childhood. Due to the poor regeneration of the articular cartilage, the collection of biopsies from juvenile cartilage is not normally justified. The diet in Kashin–Beck endemic areas has also changed due to selenium supplementation [122]. Thus, the samples collected in later life most likely do not reflect the physiological or selenium status present at the onset of the disease. Nevertheless, a recent study, which estimated the nutrient intakes of children, showed that the daily intakes of multiple nutrients, not only selenium, were lower in the selenium-supplemented Kashin–Beck area in comparison to the non-selenium-supplemented areas [123]. There were 116 nutrient-related differentially-expressed genes in the peripheral blood of Kashin–Beck disease children (51 up- and 65 down-regulated ones), and 10 significant pathways were also recognized using the KEGG and REACTOME network databases [123]. Overlapping genes with various functions were also associated with different nutrients [123]. 

## 8. Conclusions

The transcription and translation of selenoproteins require a specific and complex cellular machinery. Selenium status can define the expression levels of the selenoprotein mRNA transcripts, although there is some degree of organ- and species-dependent variation. Expressions of the non-selenoprotein genes are also affected by the selenium status, but more research is warranted to gain a deeper understanding of the interacting pathways and functions.

Recent studies, which have analysed the miRNA and lncRNA expressions and their relations, have revealed a number of them to be differentially expressed by selenium status. However, the present rather limited knowledge of the functional regulation of the cellular processes controlled by miRNAs and lncRNAs prevents strong interpretations from being made, and further studies are definitely warranted to better understand their role in the transcriptional regulation by selenium.

The Keshan and Kashin–Beck diseases have been associated with selenium deficiency, although the actual mechanisms that are behind these diseases are still not precisely understood. New findings combining microarray and proteomic analyses have revealed new ideas regarding the pathways possibly involved in these diseases.

## Abbreviations

eIF4A3	Translation initiation factor 4A3
lncRNA	Long non-coding RNA
miRNA	Micro RNA
mRNA	Messenger RNA
NMD	Nonsense-mediated decay
Nrf2	Nuclear factor, erythroid-2 like factor 2
PPAR	Peroxisome proliferator-activated receptor
SBP2	SECIS-binding protein 2
Sec	Selenocysteine
SECIS	Selenocysteine insertion sequence
tRNA	Transfer RNA
tRNA^[Ser] Sec^	Selenocysteine transfer RNA
UTR	3′-Untranslated region of messenger RNA
T3	3,5,3′-Triiodothyronine
T4	3,5,3,5″-Tetraiodothyronine
